# Effect of morphology on larvicidal activity of chemically synthesized zinc oxide nanoparticles against mosquito vectors†

**DOI:** 10.1039/d1ra00014d

**Published:** 2021-02-26

**Authors:** U. M. T. M. Gunathilaka, W. A. P. P. de Silva, S. P. Dunuweera, R. M. G. Rajapakse

**Affiliations:** Department of Chemistry, University of Peradeniya Peradeniya 20400 Sri Lanka rmgr@pdn.ac.lk; Department of Zoology, University of Peradeniya Peradeniya 20400 Sri Lanka; Postgraduate Institute of Science, University of Peradeniya Peradeniya 20400 Sri Lanka

## Abstract

We report the larvicidal effects of four different morphologies of zinc oxide nanoparticles (ZnO NPs) [star-shaped (S), needle-like (N), plate-like (P) and cubical (C)] on larvae of *Aedes albopictus* and *Anopheles vagus*; the mosquitoes causing dengue fever and malaria, respectively. The nanoparticles were characterized by several analytical techniques, and their sizes and shapes were determined. Second instar larvae of the two types of mosquitoes were exposed to several concentrations of nanoparticles (25 mg L^−1^, 50 mg L^−1^, 75 mg L^−1^, 100 mg L^−1^) at 25 ± 2 °C and 84 ± 5% R.H, separately, for each morphology. Larval mortality was reported at 24 h intervals up to 21 days. The resulting LC_50_ for *Aedes albopictus* were, respectively, 38.90 mg L^−1^, 47.53 mg L^−1^, 68.38 mg L^−1^, 50.24 mg L^−1^ for S-, N-, P- and C-shaped nanoparticles. The LC_50_ of *Anopheles vagus* is lower (LC_50_ 4.78 mg L^−1^, 6.51 mg L^−1^, 13.64 mg L^−1^, 10.47 mg L^_−1_^), respectively, for S-, N-, P- and C-shaped nanoparticles indicating that the nanoparticles are more toxic to *Anopheles vagus* larvae. The highest larvicidal effect was obtained from star-shaped nanoparticles [*Aedes albopictus* (38.90 mg L^−1^) on *Anopheles vagus* (4.78 mg L^−1^)], and the lowest was shown by the plate-like nanoparticles [*Aedes albopictus* (68.38 mg L^−1^), *Anopheles vagus* (13.64 mg L^−1^)]. The rate of development of surviving mosquito larvae was retarded when exposed to ZnO nanoparticles suggesting the possibility for these nanoparticles to kill and delay the growth of *Aedes albopictus* and *Anopheles vagus* larvae.

## Abstract 2

We report the larvicidal impacts of four different morphologies of zinc oxide nanoparticles (ZnO NPs) [star-shaped (S), needle-like (N), plate-like (P), and cubical (C)] on mosquito larvae of *Aedes albopictus* and *Anopheles vagus*.

## Graphical abstract

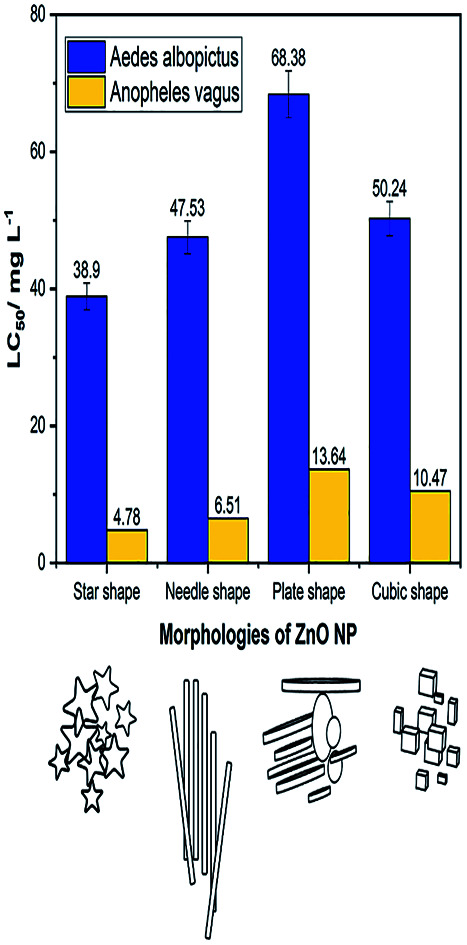

## 1. Introduction

Mosquitoes are a well-known arthropod group that transmit several fatal diseases and thereby adversely affect human health. They are widely distributed and live in almost all habitats except areas that are permanently frozen.^1^ The adult female mosquitoes can transmit disease-causing pathogens to humans and other animals through their bites when they obtain blood meals from their hosts.^2^ Malaria, Filariasis, Yellow Fever, West Nile disease, Chikungunya, Zika, and Dengue are the most common human diseases transmitted by mosquitoes.^3,4^

Dengue is a deadly mosquito-borne disease that has taken world attention due to difficulties in controlling both the disease and the vector mosquitoes.^5,6^ This disease is transmitted mainly by female *Aedes aegypti* and *Aedes albopictus mosquitoes*. Four distinct but closely related dengue virus serotypes (DEN-1, DEN-2, DEN-3, and DEN-4) have been identified.^7^ The global incidence of dengue has grown dramatically in recent decades. About half of the world's population is now at risk, with an estimated 390 million infections each year.^8^ Additionally, Yellow fever, Chikungunya, and recently recognized Zika virus (ZIKV) are also transmitted by *Aedes aegypti* and *Aedes albopictus* mosquitoes.^6,9,10^

Malaria is also one of the most widespread mosquito borne diseases in the entire world context. The female *Anopheles* mosquitoes transmit the malaria pathogen. Malaria continues to be a significant public health problem in more than ninety countries. According to the recent reports of WHO, nearly 219 million people are living in malaria-risk areas. The estimated annual mortality is in the range of 1.1 to 2.7 million deaths worldwide due to malaria.^8,11^ Out of the 422 *Anopheles* species only 70 species are considered vectors of malaria pathogen under natural conditions.^12^

Therefore, it is essential that more sophisticated and effective methods are to be developed to eliminate mosquitoes and mosquito borne diseases. In this context, increased attention has been paid towards utilizing nanotechnological techniques in controlling vector mosquitoes as a solution to emerging and re-emerging mosquito-borne diseases. According to the previous reports, nanoparticles have been used to control seven critical species of mosquitoes, including malaria vectors (*Anopheles stephensi*, *Anopheles culicifacies*, and *Anopheles subpictus*), dengue and Zika virus vectors (*Aedes aegypti* and *Aedes albopictus*), and the lymphatic filariasis vector (*Culex quinquefasciatus*).^13^ A few studies have been done to test the effect of nanoparticles on Japanese encephalitis vector *Culex tritaeniorhynchus* and the West Nile vector *Culex pipiens*.^1^ According to Vijayakumar *et al.* and Rajan *et al.*, nanoparticles of Au, Ag, Cd metals, and metal oxides of ZnO, MgO, TiO_2_, Al_2_O_3_, CuO, and CeO_2_ are found to be useful as larvicides of blood-feeding mosquitoes.^14,15^

Zinc oxide, with its impressive chemical and physical properties including high chemical stability at neutral pH, ultraviolet radiation absorption and photo-stability, and longer shelf life, is a functional, strategic, promising, and multifunctional inorganic material used in a broad range of applications in areas of optical, electrical, chemical sensors, and as a semiconductor, piezoelectric material, pyroelectric material and in solar energy conversion.^16,17^ In recent years, there has been more interest in ZnO NPs due to their large surface area to volume ratio contributing to increased chemical reactivity and catalytic activity at a lower material requirement than those of their micro and bulk counterparts.

Zinc oxide is an environmentally friendly material that is non-toxic to humans and large animals and is biocompatible. It has, therefore, been identified for its promising perspective in biological functions.^18^ For example, ZnO NPs are used as biological sensing agents, biological labelling agents, gene delivery, and drug delivery agents.^19^ Moreover, it is also recognized as antifungal and antibacterial agent.^20,21^ The use of ZnO NPs is widespread in nanomedicine, as larvicides for insect pests and vectors, and as acaricides and pediculicides and their anti-diabetic activities.^22,23^

ZnO NPs synthesized by green chemical methods have been used to determine toxic effects on mosquito larvae. For example, the larvicidal activity of ZnO NPs synthesized using *Plectranthus amboinicus* (Indian mint) leaf extract was tested against larvae of *Anopheles stephensi*, *Culex quinquefasciatus*, and *Culex tritaeniorhynchus*.^24^ Those synthesized using Momordica charantia leaf extract were tested against the fourth instar larvae of *Culex quinquefasciatus* and *Anopheles stephensi*.^25^ ZnO NPs synthesized using a brown algal species, *Sargassum wightii* extracts, were also reported for the efficacy in controlling *Anopheles stephensi* larvae and pupae.^26^ Leaf extracts of *Terminalia chebula* (Gall nut) also reported larvicidal activities against *Culex quinquefasciatus*, *Anopheles stephensi*, and *Aedes aegypti*.^27^ In addition to green-synthesized ZnO NPs, those synthesized chemically were also used to investigate larvicidal activity against *Anopheles subpictus*, *Culex quinquefasciatus* and *Aedes aegypti* larvae.^28,29^

Some studies reported the mechanism of the larvicidal activity of ZnO NPs. According to Benelli *et al.* and Banumathi *et al.*, ZnO NPs causes several morphological and histological abnormalities, including shrinkage in the abdominal region, thorax shape changes, midgut damages, loss of lateral hairs, anal gills and brushes, and accumulation of ZnO NPs in the thorax and abdomen in larvae of *Aedes aegypti*.^13,30^ Despite the enormous studies in this field, no consensus is achieved about the morphology of nanoparticles for mosquito larval toxicity.^31^

To fill in this gap, we investigated the impact of the morphology of ZnO nanostructures towards the dengue vector mosquito of *Aedes albopictus* and potential malaria vector of *Anopheles vagus.* Accordingly, mosquito larvae were exposed to synthesized ZnO NPs prepared using wet-chemical and hydrothermal synthesis methods, having four distinctive shapes. Monge *et al.* has clearly expressed the main factors affecting the shapes of ZnO NPs. Namely the nature of the ligand, the concentration of the different reagents, surfactants and the solvent used, the total concentration of the reagents, the experimental reaction time, reaction medium, pH and temperature. Moreover, different ligands (initial zinc salts) and surfactants present in the solution can also affect the particle size and shape.^32^ Growth inhibition and mortality were measured as toxicity responses. This is the first-time report concerning comparing the larvicidal effects of ZnO NPs with the shape-dependent toxicity against *Aedes albopictus* and *Anopheles vagus* mosquitoes. We explain the results obtained using possible mechanisms of action of ZnO NPs of different shapes.

## 2. Results and discussion

### 2.1 Morphological and structural properties of ZnO NPs

**Fig. 1:**
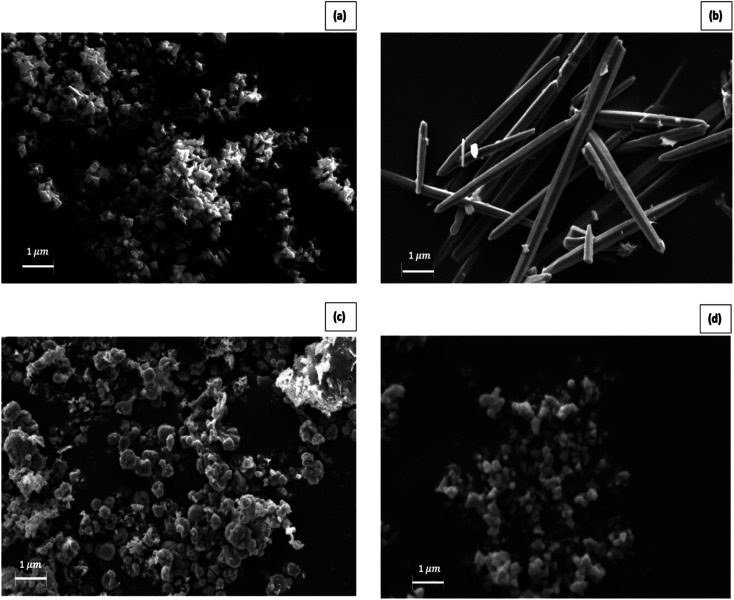
SEM images of materials prepared by (a) method 1, (b) method 2, (c) method 3 and (d) method (4).

The SEM images of the materials prepared by methods 1, 2, 3, and 4 are shown in Fig. 1(a), (b), (c) and (d), respectively. The images clearly show star-shaped, needle-like, plate-like, and cubic-shaped nanoparticles in the respective samples.

Fig. S1(a)–(d)† show the XRF spectra of the four different morphologies of the products synthesized. The spectra contain peaks at 8.6 keV and 9.6 keV, which are characteristic Zn (Kα) in Zn (Kβ) peaks reported by Roy *et al.*^33^ Unfortunately, our XRF instrument is incapable of measuring elements of an atomic number less than 13. Therefore, the results indicate that the products formed are free of impurities containing the element with an atomic number higher than 13.

As evident from the EDX spectra shown in Fig. S2(a)–(d),† all four morphologies of materials consist of only zinc (Zn) and oxygen (O) as prominent peaks thus proving that the materials prepared in all four methods are indeed ZnO.

The controlled precipitation of Zn^2+^ and OH^−^ ions gives Zn(OH)_2_ nanoparticles. When Zn(OH)_2_ nanoparticles are annealed, they undergo dehydration at 500 °C, for 2 h, forming ZnO nanoparticles. Interestingly, the type of Zn^2+^ and OH^−^ precursors, the presence or absence of surfactant ions (cetyltrimethylammonium ions) with or without autoclaving, give rise to different morphologies of Zn(OH)_2_ nanoparticles, which can subsequently be dehydrated to get shape-specific ZnO nanoparticles. When the surfactant is used above its critical micelle concentration, micelles are formed in the solution. These micelles act as soft-templates supporting to nucleate in their core. The nuclei thus formed grow in the spaces between micellular surfactant molecules to result in the growth of star-shaped nanoparticles during hydrothermal treatment.

When there is no surfactant in the medium, the growth under hydrothermal conditions gives a needle-shaped in an ethanolic medium. The conditions of hydrothermal treatment favour the elongation of particles in one dimension. Ethanolic medium is preferred over aqueous medium since zinc hydroxide particles formed readily undergo dehydration to be converted to ZnO NPs.

**Fig. 2:**
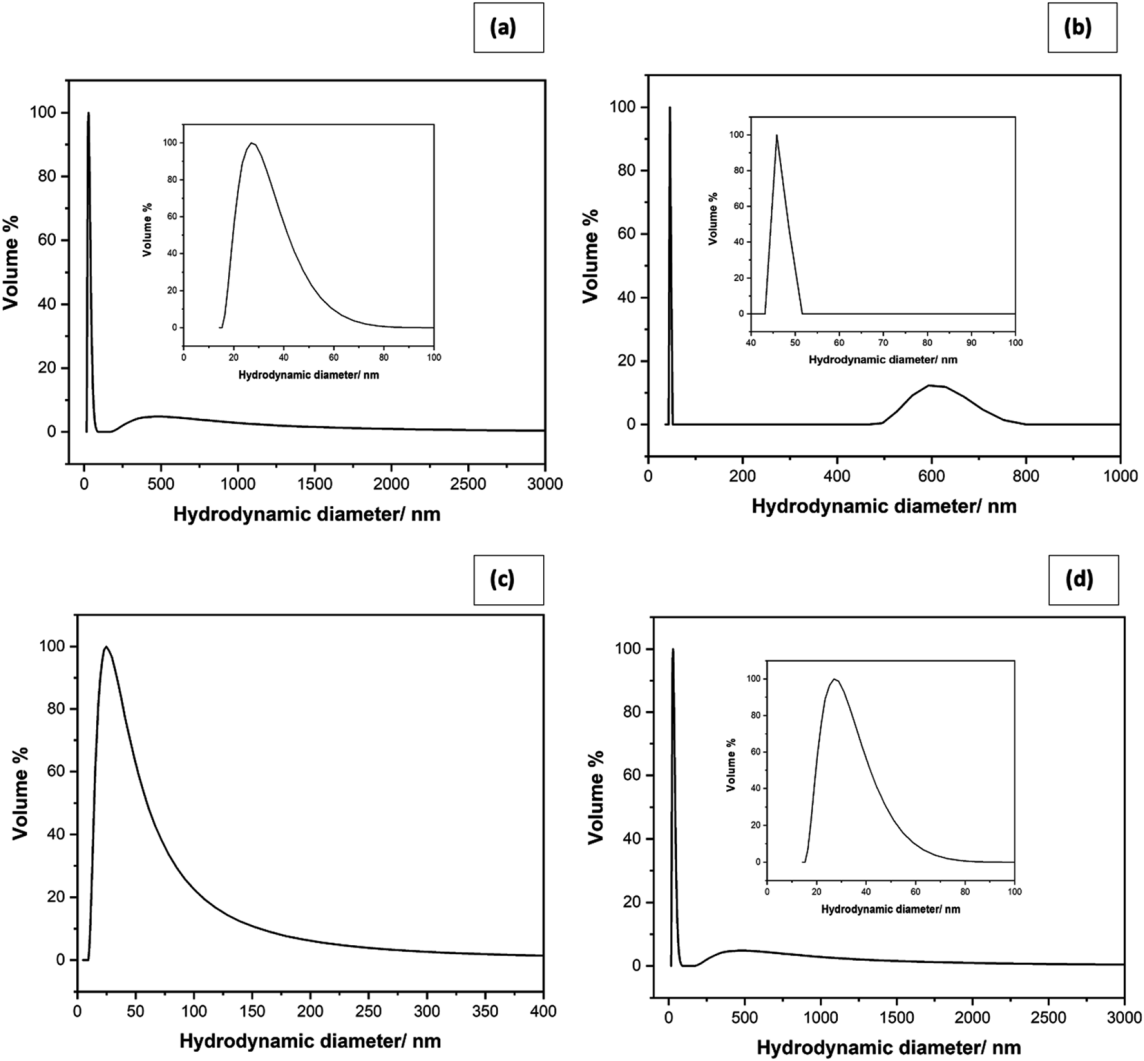
Particle size distribution of (a) star shaped, (b) needle-like, (c) plate-like and (d) cubic-shaped ZnO NPs.

Fig. 2 shows the particle size analysis results. The h *y*-axis of the plot represents the parameter called, Q3% which gives the percentage of particles with a given hydrodynamic radius. For the star-shaped ZnO NP (Fig. 2(a)), the plot contains a narrow band with an average particle size of 27.0 nm in size range from 21.8 nm to 47.6 nm. Another band is bounded by 371.8 nm and 788.3 nm for average particle size 458.6 nm at the 90% confidence interval. When the sizes are compared with those shown in the SEM image, it is clear that the small size of 27 nm is due to the somewhat spherical central core diameter while the sub-micrometer size around 458 nm is due to plates of the stars. As shown in Fig. 2(b), the synthesized needle-like colloidal solution contains particles in size range from 43.1 nm to 48.7 nm with an average particle size of 45.8 nm. As shown in the SEM images, 45 nm size is due to the diameter of the needles. When an aqueous medium is used, the shape of nanoparticles obtained depends on the strength of the base used and the counter ion present. When NaOH is used as the base and nitrate ions are present as counterions, the preferred morphology of zinc hydroxide particles is plate-like. However, when aqueous ammonia is used in acetate counter ions, cuboidal nanoparticles are formed. The ready adsorption of acetate ions on the surfaces of nanoparticles during slow growth of results in a more symmetrical structure leading to the cuboidal shape.

Moreover, a second band shows an average size of 593.3 nm in a 90% confidence interval, which is due to the length of the needles. Fig. 2(c) shows the particle size distribution of plate-like ZnO NPs with an average particle size of 24.9 nm in 90% confidence interval. It lies in between 21.2 nm and 50.0 nm range with a continuous tail extending towards 247.9 nm. Unlike previous shapes that gave two distinct bands, the plate-shaped gives one highly asymmetric band with a mean in the 24.9 nm and extending way into larger sizes. This may be due to the polydispersity of the material prepared in the aqueous solution. Fig. 2(d) shows the particle size distribution of cubic-shaped ZnO NPs with the average particle size as 24.9 nm with a higher percentage of 90% confidence interval.

**Fig. 3:**
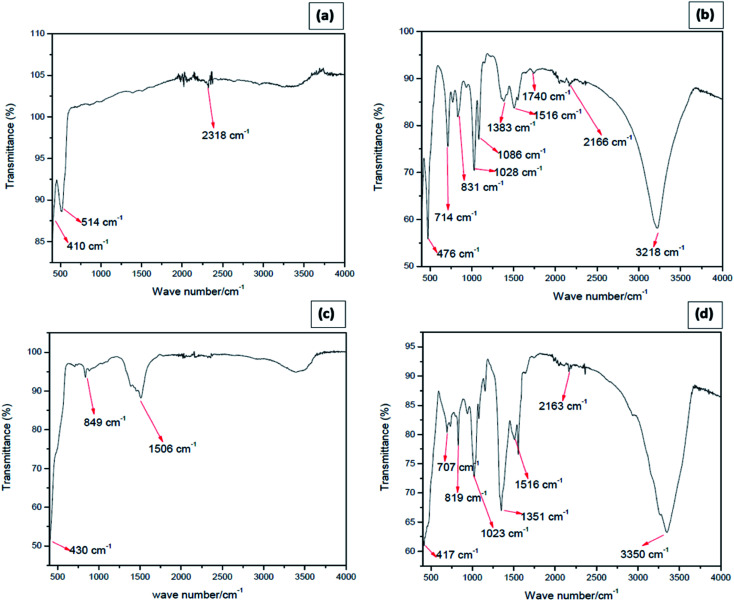
FTIR spectrum of (a) star-shaped, (b) needle-like, (c) plate-shape and (d) cubic zinc oxide nanoparticles.

As evidenced from the FT-IR spectrum of the star-shaped NPs, provided in Fig. 3(a), the absorption band at 410 cm^−1^ and 514 cm^−1^ are due to Zn–O vibrations, which confirms the presence of the ZnO.^34^ The functional group of CO_2_ at the wavenumber of 2318 cm^−1^ is predicted to come from the outside air, as reported in the literature.^35^ The absence of N–O vibrations suggests that nitrate ions are not adsorbed on star-shaped ZnO nanoparticles. The washing procedure adapted has removed any adsorbed surfactant molecules since no detectable bands correspond to C–C, C–H, C–N, and N–H vibrations. The FTIR spectrum of needle-like NPs (Fig. 3(b)) shows the broadband at 3218 cm^−1^ due to the C–H stretching of an alkenyl group, and the band obtained at 2166 cm^−1^ is due to the presence of atmospheric CO_2_.^36^ The band obtained at 1740 cm^−1^ corresponds to the C=O stretching of the functional group. The carboxyl C–O bond was also identified at the wavenumber of 1086 cm^−1^, 1383 cm^−1^, and 1516 cm^−1^, which may be due to adsorbed acetate ions on the nanoparticle surfaces.^38^ The absorption band at 3218 cm^−1^ is likely due to O–H stretching, and that at 1028 cm^−1^ may be due to C–O vibrations of adsorbed ethanol molecules. The Zn–OH functional group appears at 831 cm^−1^. Zinc oxide is known to adsorb moisture forming surface hydroxyl groups, and the band could be due to those adsorbed water on ZnO nano-needles. The narrowband at 476 cm^−1^ represents the availability of ZnO in the prepared sample.^34^Fig. 3(c) shows the FTIR spectrum of plate shape ZnO NPs. The absorption band at 430 cm^−1^ is the characteristic absorption of the Zn–O bond, and the absorption band at 849 cm^−1^ represents Zn–OH stretching.^34^ The broad absorption band at 1506 cm^−1^ can be attributed to the characteristic absorption of nitride since zinc nitrate was used as a precursor for the preparation of plate-like ZnO nanoparticles.^38^ According to the FTIR spectrum of cubic shape ZnO NPs (Fig. 3(d)), Zn–O functional group is identified at the wavenumbers of 417 cm^−1^, which confirms the formation of ZnO. Zn–OH functional group appears at 819 cm^−1^.

Furthermore, at 3350 cm^−1^, the hydroxyl stretching vibration of O–H often occurred. The appearance of the O–H bond showed the existence of water absorbed in the ZnO particles surface, which gives evidence by Laurenti *et al.*^35^ The carboxyl C–O bond was also identified at the wavenumber of 1351 cm^−1^ and 1516 cm^−1^, which may be due to adsorbed acetate ions on the nanoparticle surfaces.^37^

Except for unique vibrational bands corresponding to Zn–O, FTIR spectra show bands of different functional groups due to the residues adsorbed onto ZnO NPs; particularly, the anions of the precursor salts used. Even though the Zn^2+^ ions show toxic action to living cells, acetate and nitrate ions do not affect larvicidal activity.^39^ Hence, it is evident that the differences in larvicidal activity are basically due to the morphological differences of the ZnO NPs. ZnO NPs are known to destroy bacteria by five modes of actions. Among these, Zn^2+^ ion toxicity and mechanical action are the predominant modes. Morphologies with sharp edges such as stars and needles can damage cell walls bacteria and small animals just as the way damaging by scissors (*vide infra*).^40^

### 2.2 Effects of four different morphologies of ZnO NPs on second instar stage mosquito larvae of *Aedes albopictus* and *Anopheles vagus*

Percentage mortality values were calculated for a 24 h interval for 21 days. Using percentage mortality values (calculated using eqn (1)) and sample concentrations of ZnO NPs, LC_50_ values were calculated to determine the toxic effect of the four different morphologies of ZnO NPs.1
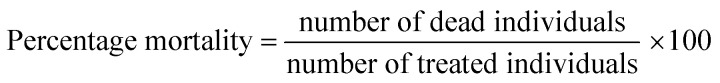


Chemically synthesized ZnO NPs showed noticeable larvicidal effects against the second instar stage of *Aedes albopictus* and *Anopheles vagus* mosquito larvae. Tables S1 and S2† summarize the results of the larval bioassays of *Aedes albopictus* and *Anopheles vagus.* Compared to the other morphologies of ZnO nanomaterial, star-shaped NPs' showed the highest toxicity against both mosquito larvae with the lowest LC_50_ values of 38.90 mg L^−1^ for *Aedes albopictus* and 4.78 mg L^−1^ for *Anopheles vagus.* Second highest larvicidal effect was shown by the needle-like ZnO NPs with LC_50_ 47.53 mg L^−1^ and 6.51 mg L^−1^, respectively, for *Aedes albopictus* and *Anopheles vagus.* The cubic-shaped ZnO NPs show LC_50_ value of 50.24 mg L^−1^ for *Aedes albopictus* and 13.64 mg L^−1^ for *Anopheles vagus.* The results confirmed that plate-like ZnO NPs have the lowest toxicity with the LC_50_ value of 68.38 mg L^−1^ and 10.47 mg L^−1^, respectively, for *Aedes albopictus* and *Anopheles vagus.*

Similarly, many studies have confirmed the lethal effect of ZnO NPs on Aedes mosquito larvae. The majority of those studies have used the primary dengue vector, *Aedes aegypti*, as the testing organism, and the ZnO NP's have been synthesized by green synthesis. Thus, we are not able to directly compare our results with the previous findings. Larvicidal activity of green synthesized ZnO nanorods obtained from Myristica fragrans leaf extracts (Nutmeg) against *Aedes aegypti* was reported by Ashokan *et al.*^23^ Their study has obtained LC_50_ values of 3.44 mg L^−1^ (larva I) and 14.63 mg L^−1^ (pupa), which are significantly low compared to the LC_50_'s of our study. Similarly, ZnO NPs synthesized by leaf extracts of wild tobacco (leschena ultiana) also have shown high toxicity against larvae of *Aedes aegypti* with the LC_50_ value 1.57 mg L^−1^.^30^ The apparent larvicidal activity of ZnO NPs on dengue mosquito larvae was further proved by the Naif *et al.*^41^ Their study reported the toxic effect of green synthesized spherical shape ZnO NPs obtained from leaf powder of Blood lily (Scadoxus multiflorus) against *Aedes aegypti* larvae and eggs with the LC_50_ value of 34.04 mg L^−1^ and 32.73 mg L^−1^, respectively.

Several studies have reported the larvicidal activity of ZnO NP's on *Anopheles stephensi* mosquito larvae. For instance, Vijayakumar *et al.* reported that ZnO NPs synthesized from leaf extracts of *Plectranthus amboinicus* (Indian mint or Kapparawalliya) are lethal to *Anopheles stephensi* larvae.^24^ The reported LC_50_ value was 3.11 mg L^−1^. Their study has shown that ZnO NP's can damage cells and tissues of the larval mid-gut. Similarly, spherical ZnO NPs synthesized by chemical methods and synthesized from leaf extracts of *Momordica charantia* (Bitter gourd) and *Sargassum wightii*, one of the marine brown algal species in tropical belts reported to have lethal effects against larvae of *Anopheles stephensi*.^25,26,29^ The LC_50_ values reported from those studies are 11.14 mg L^−1^, 5.42 mg L^−1^, and 4.330 mg L^−1^, respectively.

**Fig. 4:**
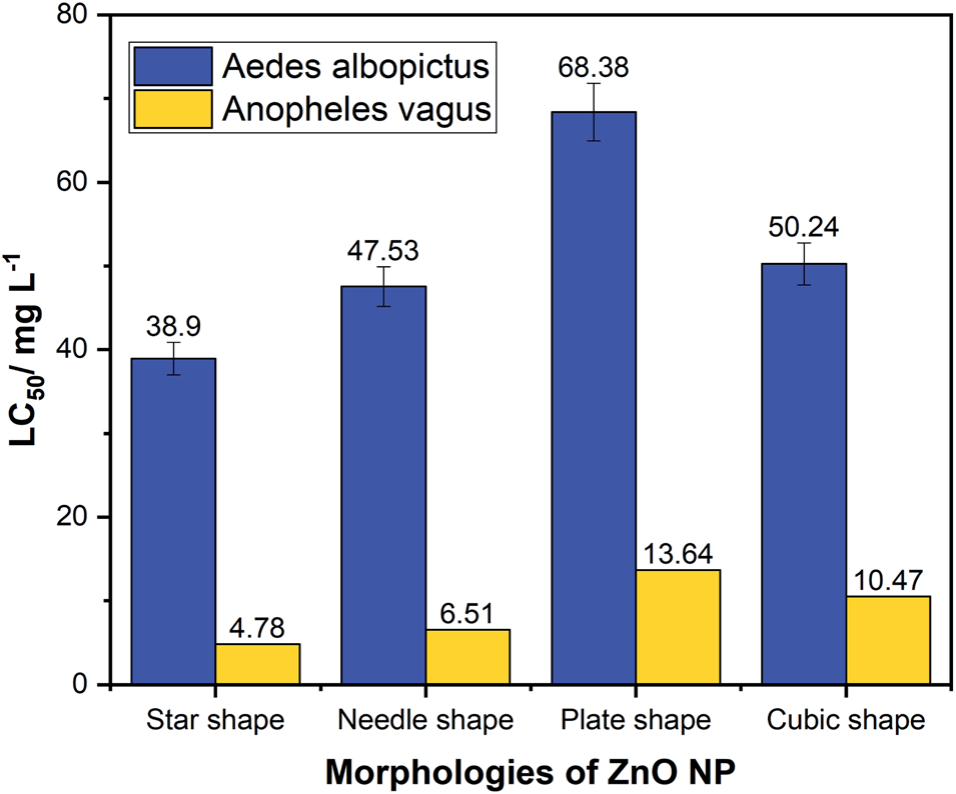
Comparison of LC_50_ values of four different morphologies of zinc oxide nanoparticles against larvae of *Aedes albopictus* and *Anopheles vagus* mosquitoes.

The results showed that the possible malaria vector *An. vagus* mosquito larvae are more responsive to ZnO NPs than to larvae of the secondary dengue vector *Aedes albopictus* (Table 1 and Fig. 4). Although our study and the previous studies have also been identified more chemical resistance to the ZnO NPs by the *Aedes* other than *Anopheles* mosquito larvae, the detailed studies on how exposure to ZnO can affect different mosquito larval species have not been studied yet. Therefore, the toxicity mechanisms to different mosquito larval species with the ZnO NPs needed to be investigated.

**Table 1:** Comparison of the toxicity effect of ZnO NP's on *Aedes albopictus* and *Anopheles vagus*^a^

Shape of nanoparticles	LC_50_ value (mg L^−1^)		*T*-Test value	*P*-Value
	*Aedes albopictus*	*Anopheles vagus*		
Star shape	38.90	4.78	4.19	0.024
Needle shape	47.53	6.51	6.45	0.007
Plate shape	68.38	13.64	23.33	0.0001
Cubic shape	50.24	10.47	8.05	0.004

a LC_50_: lethal dose which kills 50% of the exposed larvae, significant level *α* = 0.05.

We observed a significant difference between the toxicity of ZnO NP's on these two different larval species (*p* < 0.05). Among the four morphologies, the star-shaped nanoparticles showed the least LC_50_ for both mosquito larvae as 38.9 mg L^−1^ and 4.78 mg L^−1^ for *Aedes albopictus* and *Anopheles vagus*, respectively, suggesting that star shape is the most effective morphology among four tested types of ZnO NPs. The plate-shaped ZnO NPs have the lowest effectiveness against both mosquito larvae, with LC_50_ of 68.38 mg L^−1^ for *Aedes albopictus* and 13.64 mg L^−1^ for *Anopheles vagus*.

### 2.3 Effect of four different morphologies of ZnO NPs on the growth of *Aedes albopictus* and *Anopheles vagus* mosquito larvae

**Fig. 5:**
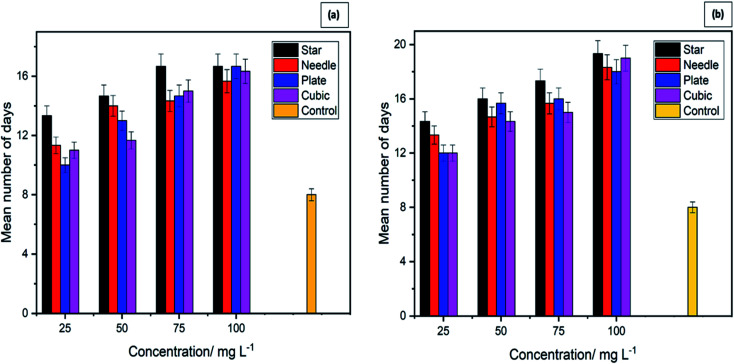
Duration in days required by mosquito larvae to complete the life cycle when exposed to different morphologies and concentrations of zinc oxide nanoparticles. (a) *Aedes albopictus*, (b) *Anopheles vagus*.

Mosquito larvae, which have survived after exposure to different shaped ZnO NPs for 24 h, were continuously monitored for 21 days to determine whether there are any effects of ZnO NP's on the development of survived larvae. Results revealed the delays in developing the surviving larvae after they were exposed to four morphologies of ZnO NPs. These results are depicted in Fig. 5(a) and (b) and Table 2. These results provide strong evidence for the dose-dependent effect of ZnO NPs on the development of mosquito larvae. Larvae in the control experiment emerged as adults within 8 ± 1 days.

**Table 2:** Duration in days for the development of *Aedes albopictus* and *Anopheles vagus* mosquito larvae with different morphologies and concentrations of ZnO NPs

Concentration (mg L^−1^)	Duration in days (mean ± standard error) for the development of larvae							
	*Aedes albopictus*				*Anopheles vagus*			
	Star	Needle	Plate	Cubic	Star	Needle	Plate	Cubic
25	13.33 ± 0.33	11.33 ± 0.33	10.00 ± 0.57	11.00 ± 0.57	14.33 ± 0.33	13.33 ± 0.33	12.00 ± 0.57	12.00 ± 00
50	14.67 ± 0.33	14.00 ± 0.57	13.00 ± 0.57	11.67 ± 0.33	16.00 ± 00	14.67 ± 0.33	15.67 ± 0.33	14.33 ± 0.33
75	16.67 ± 0.33	14.33 ± 0.88	14.67 ± 0.33	15.00 ± 0.57	17.33 ± 0.33	15.67 ± 0.33	16.00 ± 00	15.00 ± 0.57
100	17.00 ± 00	15.67 ± 0.67	16.67 ± 0.33	16.33 ± 0.33	19.33 ± 0.33	18.33 ± 0.33	18.00 ± 0.57	19.00 ± 00

In contrast, larvae in the treatment containers took more than 8 days to complete the larval instars. The number of days that they spend as larvae and to emerge as adults varied with the exposure concentration. The larvae exposed to the highest concentration of NPs took the most prolonged time period to complete the life cycle providing strong evidence of the effect of ZnO NPs on the larval development. Similar results were shown by both larval species, further strengthening the impact of ZnO NPs on the rate of growth of mosquito vectors.

### 2.4 Mechanisms and structure dependence of ZnO NPs for its larvicidal activity

In our studies, we have synthesized nano-sized ZnO NPs. According to Moos *et al.*, it has been reported that nano-sized ZnO NPs showed more cytotoxicity than the micro-sized ZnO NPs with LC_50_ values of 15 and 29 μg cm^−2^, respectively.^42^ Kocbek *et al.* (2010) proved that prolonged exposure to ZnO NPs results in poor mitochondrial activity, loss of natural cell morphology, and disturbances in cell cycle distribution.^43^ According to Olejnik *et al.*, rod-shaped ZnO NPs show higher toxicity than spherical particles emphasizing the significance of both particle size and shape for cytotoxicity.^44^ Yang *et al.* have shown that zinc oxide rods and wires can penetrate through bacteria more readily than spheres.^45^ Talebian *et al.* have shown flower-shaped ZnO NPs have higher biocidal activity than the ZnO NP spheres and rods.^46^

Mir *et al.* have clearly explained ZnO NP structure and larvicidal activity.^47^ ZnO NP induced morphological changes in five hemocyte types have been discussed. When hemocytes are exposed to ZnO NPs, various structural deformations and cell membrane disruptions were reported. Moreover, hemocyte cell viability was significantly decreased when they are exposed to ZnO NPs. Hence the physical morphology and structure are shown to contribute to the mechanisms of larvicidal activity.^44^

Most of the scientific findings on ZnO NPs cytotoxicity and larvicidal activity explain a common mechanism. Briefly, ZnO NPs can accumulate inside the cytoplasm by disintegrating the cell membranes. It releases cytotoxic Zn^2+^ ions to provoke cell apoptosis. The soluble fraction of the ZnO NPs at the equilibrium (Zn^2+^ ion) exerts a higher toxic effect.^48^ Zn^2+^ ion dissolution behaviour and effectiveness of cellular uptake depend on many factors, including the physicochemical properties (size and shape of the particle, surface area) or intercellular physical environment (cell type, pH, temperature) ZnO NPs can trigger reactive oxygen species (ROS) production inside the cytoplasm which can induce cytotoxicity^40,49^ They can damage cells by oxidizing lipids and disrupting structural proteins, enzymes, and nucleic acids. ZnO NPs has induced programmed cell death (apoptosis) in larval hemocytes.^47^

Comparing the effects of ZnO NPs with different shapes (star, needle, plate, and cubic) on *Aedes albopictus* and *Anopheles vagus* larvae have shown that star and needle shape NPs cause death of a larger proportion of larvae than cubic and plate shape NPs. According to the literature evidences this experimental outcome might be due to the high facet diversity on the star-shaped ZnO NPs. Even though ZnO NPs with different morphologies have different active facets, a few studies have evaluated the facet-dependence of ZnO NPs.^50^ When the facets of the ZnO NPs surface become complicated and diverse, the larvicidal activity has increased. Furthermore, the facets provide a higher number of polar surfaces, and high surface to volume ratio^51^ to trap more oxygen molecules. Vacancies filled with oxygen molecules can be efficiently utilized by the cells to generate ROS, enhancing larvicidal activity.^52^ This is further proven by the capacity of star-like and needle-like NPs for damaging cells and tissue upon direct contact.^53^

The other possible reason is the surface defects of different ZnO NPs structures, which strongly influence the larvicidal activity. Surface defects at the edges and corners can be abrasive, damaging the cell membrane of larvae.^54^ Star-shaped structure has randomly oriented spatial distribution of spikes, which can be more destructive than other structures. Moreover, the shape of star and needle NPs may increase the larvicidal activity by cutting the larval body, blocking respiratory systems, and damaging the midgut.^13,30^

## 3. Experimental section

### 3.1 Preparation of ZnO NPs

All the chemicals used were of analytical grade, purchased from Sigma-Aldrich, and were used without further purification. The ZnO NPs were prepared from four different methods utilizing solution-phase synthesis with and without autoclaving.

In the method 1, 0.595 g Zn(NO_3_)_2_·6H_2_O and 0.050 g of cetyltrimethylammonium chloride (CTAC) were dissolved in 20.00 mL absolute ethanolic solution by stirring for 2 h. Also, 0.200 M of NaOH in ethanol was prepared by dissolving 0.200 g of NaOH in 25.00 mL of alcoholic solution. The two precursor solutions were mixed well and allowed to react by stirring for 4 h. The reaction mixture was autoclaved in a Teflon-lined autoclave at 140 °C for 10 h. The suspension thus obtained was centrifuged and filtered. The solid product was washed with ethanol and subsequently with distilled water. It was then oven-dried at 75 °C for 24 h and annealed in a furnace at 500 °C for 2 h.

In method 2, 20.00 mL of 0.100 M Zn^2+^ in absolute ethanol was prepared by dissolving 0.439 g of Zn(O_2_CCH_3_)_2_·(H_2_O)_2_ ethanol by stirring for 2 h. To the solution thus obtained, 25.00 mL of 0.500 M of ammonia solution was added in a dropwise manner while stirring for 4 h. After completing the reaction, the reaction mixture was autoclaved in a Teflon-lined autoclave at 140 °C for 10 h. The suspension thus obtained was filtered, and the solid product obtained was washed with ethanol and distilled water. It was oven-dried at 75 °C for 24 h and subsequently annealed in a furnace at 500 °C for 2 h.

In method 3, 2.975 g of Zn(NO_3_)_2_·6H_2_O was dissolved in distilled water to make 100.00 mL of a 0.100 mol dm^−3^ solution. To this solution, 100.00 mL of 0.200 mol dm^−3^ aqueous NaOH solution was added dropwise under constant stirring for 2 h. The suspension obtained was then centrifuged and filtered. The solid product was washed with distilled water. It was oven-dried at 75 °C for 24 h and subsequently annealed in a furnace at 500 °C for 2 h.

In method 4, aqueous solutions of Zn(O_2_CCH_3_)_2_·(H_2_O)_2_ and NaOH was reacted in a wet chemical synthesis method. To do so, 2.195 g of Zn(O_2_CCH_3_)_2_·(H_2_O)_2_ was dissolved in distilled water to obtain a 100.0 mL of 0.100 mol dm^−3^ solution, and 0.800 g of NaOH was dissolved in distilled water to obtain 200.0 mL of 0.100 mol dm^−3^. The NaOH solution was added dropwise to the Zn^2+^ solution under continuous stirring for 3 h. The residue obtained was separated by filtration, and the solid was washed with distilled water. The solid product was oven-dried at 75 °C for 24 h and subsequently annealed in a furnace at 500 °C for 2 h.

### 3.2 Characterization analysis

The materials prepared were characterized by SEM (EVO LS15 OXFORD X-act Scanning Electron Microscope with the aid of an Au-coated powdered sample) to determine their morphologies. Their purities were analyzed by EDX and X-ray fluorescence (XRF, Fisher Instruments) studies. Laser light scattering-based particle size analysis (CILAS Particle Size Analyzer NANO DS) were performed to determine their size distributions. FT-IR (Shimadzu IR-Prestige 21 Instrument) spectroscopic studies were done with the KBr pellet method.

### 3.3 Rearing of mosquitoes

*Aedes albopictus* and *Anopheles vagus* mosquito larvae were collected from the premises of the University of Peradeniya (7.26° N, 80.59°), Upper Hanthana area (7.16° N, 80.38′), and Uda Peradeniya area (7.15° N, 80.36° E) of the Kandy district, Sri Lanka. Those larvae were kept in the insectary of the Department of Zoology, University of Peradeniya, and reared in plastic trays (30 cm × 25 cm × 5 cm) with de-chlorinated tap water under laboratory conditions (25 ± 2 °C, 84 ± 5% relative humidity). Powdered fish food was supplied twice a day for mosquito larvae as the larval food source. Adult and larval mosquitoes were identified to species level using standard taxonomic keys.^55^ Only the second instar larvae were used for bioassay experiments.

### 3.4 Toxic effect of nanoparticles on *Aedes albopictus* and *Anopheles vagus* larvae

The toxicity test of synthesized four morphologies of ZnO NPs was performed by placing 10 mosquito larvae into small disposable plastic test cups containing nanoparticles and 200 mL of distilled water. Firstly, the 0.1% stock solution was prepared by dissolving 20 mg of four morphologies of ZnO NPs with 20.00 mL of distilled water. Test concentrations (100, 75, 50, 25 mg L^−1^) were obtained by adding appropriate volumes of stock solutions to the small disposable plastic test cups, diluted with required amounts of distilled water to make 200.0 mL volume. Each test included a set of control groups (distilled water) with three replicates for each concentration for four different shapes of nanoparticles (star, plate, needle, and cubic shapes). Larval mortalities were recorded after 24 h exposure to each test solution. The observations were made until 21 days in each sample to determine the larval toxicity of each of the four powders on the second instar larvae of *Aedes albopictus* and *Anopheles vagus*. Also, the mortality of different morphologies of ZnO NPs on survived larvae was monitored for 21 days to observe variations in the growth patterns.

### 3.5 Statistical analysis

Mortality data were used to determine the LC_50_ values (Lethal dosage, which kills the 50% of the mosquito larvae tested) of each morphotype on each species of mosquito larvae. LC_50_ values were calculated from a log dosage–probit mortality regression line using SPSS version 18 software program.^56^ Additionally, the standard deviation and confidence intervals of the means of LC_50_ values were calculated, and the validity of the test series was concluded using relative standard deviation (or the coefficient of variation). The significant difference between the groups of four nanoparticles was calculated using the *t*-test using Minitab 17. Statistical significance was accepted at a level of *p* < 0.05.

## 4. Conclusions

In this study, we investigated the possibility of nanotechnology in controlling two mosquito vectors causing devastating mosquito-borne diseases. The larvicidal effect of ZnO NPs on the potential malaria vector *Anopheles vagus* and the secondary dengue vector mosquito, *Aedes albopictus*, were evaluated. Four different morphologies of ZnO NPs were able to prepare successfully using solution-phase and hydrothermal chemical synthesis methods with and without a surfactant. EDX, FTIR, SEM, and XRF studies of synthesized nanoparticles provide evidence of the successful synthesis of ZnO NPs. SEM images showed that these nanoparticles are in four different sizes and shapes. DLS studies reveal that all the synthesized zinc oxide particles are in the nano-range.

The larvicidal assays confirmed that *Anopheles vagus* mosquito larvae are more vulnerable to ZnO NPs than that of *Aedes albopictus* larvae. Star-shaped nanoparticles had the lowest LC_50_ value ensuring that it is the most effective as a larvicide morphology among the four types used. Percentage mortality of mosquito larvae depends on both concentration of the nano-solution and the shape of the ZnO NPs. The star-shaped and needle-like nanoparticles of ZnO could be considered potentially promising and effective morphologies for mosquito larvicidal activities than that of the plate and cubic shapes ZnO NPs.In the presence of ZnO NPs, the rates of development of both mosquito larvae were comparatively low.

Therefore, the study results also proposed that ZnO NPs can delay the growth of *Aedes albopictus* and *Anopheles vagus* mosquito larvae. Even though further studies are needed to confirm the mechanism that affects the mortality of mosquito larvae when they are exposed to different morphologies of ZnO NPs, we provide vital information about the shape-dependent toxicity of chemically synthesized ZnO NPs. Overall, the study results propose ZnO NPs as an alternative approach to current mosquito control practices with less harmful effects on ecosystems.

## Author contributions

U. M. T. M. Gunathilaka: Chemical analyses, Data analyses, visualization, Data curation, Methodology, interpretation, Writing – original draft. W. A. P. P. de Silva: Conceptualization, Resources, Project administration, Supervision, Review & editing, Funding acquisition, and Finalized the final version of the manuscript. S. P. Dunuweera: Preliminary data interpretation, Visualization, draft preparation, Designed the final manuscript structure. R. M. G. Rajapakse: Conceptualization, Project administration, Supervision, Resources, Writing, review & editing, Funding acquisition, and Finalized the final version of the manuscript.

## Conflicts of interest

There are no conflicts to declare.

## Supplementary Material

RA-011-D1RA00014D-s001
