# Supplementary material for: Effect of morphology on larvicidal activity of chemically synthesized zinc oxide nanoparticles against mosquito vectors
Source: RSC Adv. 2021 Feb 26;11(15):8857–66. doi: 10.1039/d1ra00014d (PMC8695257; doi:10.1039/d1ra00014d)
Supplement: RA-011-D1RA00014D-s001 [file RA-011-D1RA00014D-s001.pdf]

Electronic Supplementary Material (ESI) for RSC Advances.
This journal is © The Royal Society of Chemistry 2021

Supporting information

Effect of Morphology on Larvicidal activity of chemically synthesized zinc oxide nanoparticles against mosquito vectors

U.M.T.M Gunathilaka, [a,b,c] W.A.P.P.de Silva,[b,c] S.P. Dunuweera, [a,c] and R.M.G. Rajapakse *[a,c]

[a]Department of Chemistry, University of Peradeniya, Peradeniya 20400, Sri Lanka.

[b]Department of Zoology, University of Peradeniya, Peradeniya 20400, Sri Lanka.

[c]Postgraduate Institute of Science, University of Peradeniya, Peradeniya 20400, Sri Lanka.

Table of contents

Figure.S1: XRF report of zinc oxide nanoparticles. (a) Star shape, (b) Needle shape, (c) Plate shape, (d) Cubic shape

Figure.S2: EDX spectrum of zinc oxide nanoparticles. (a) Star shape, (b) Needle shape, (c) Plate shape, (d) Cubic shape

Table S1: Mean mortality of *Aedes albopictus* mosquito larvae when exposed to different concentrations of four different morphologies of ZnO NPs

Table S2: Mean mortality of *Anopheles vagus* mosquito larvae when exposed to different concentration of four different morphologies of ZnO NPs

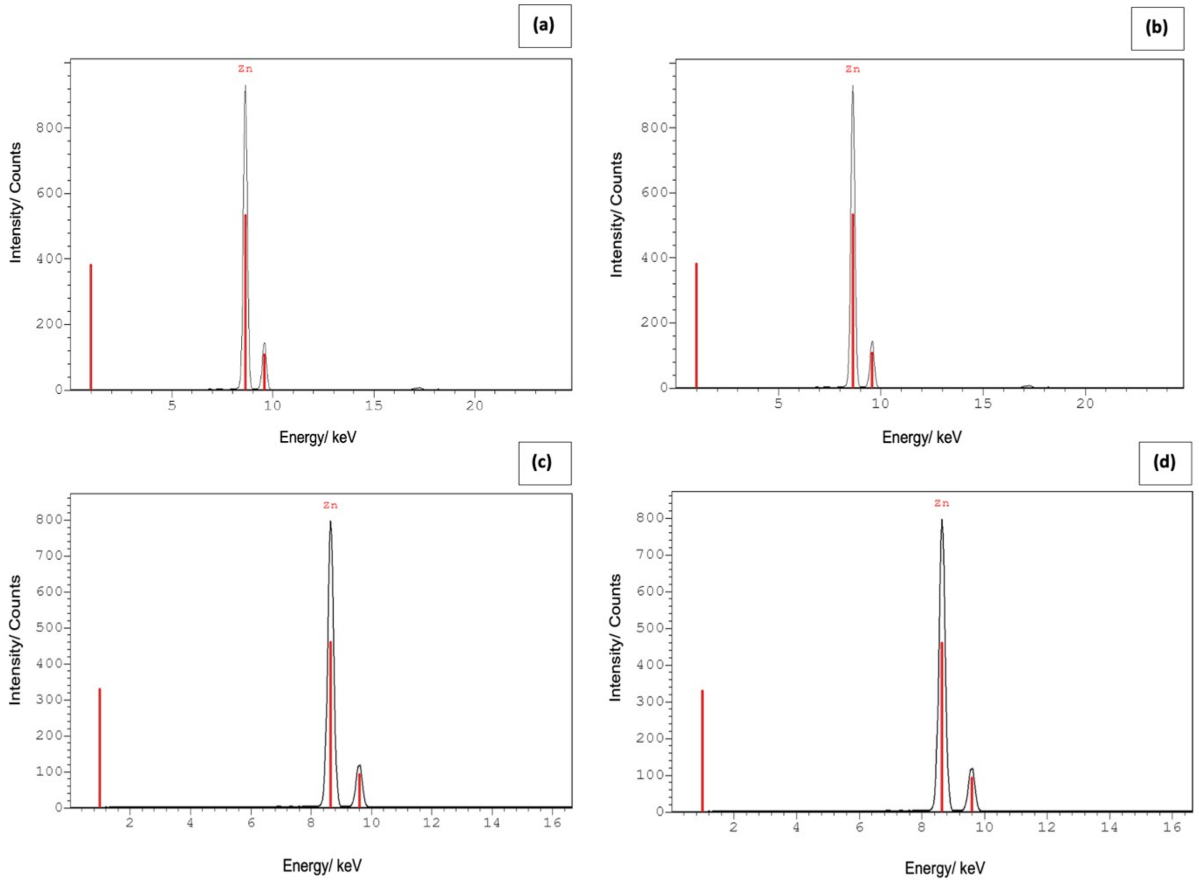

Figure.S1: XRF report of zinc oxide nanoparticles. (a) Star shape, (b) Needle shape, (c) Plate shape, (d) Cubic shape

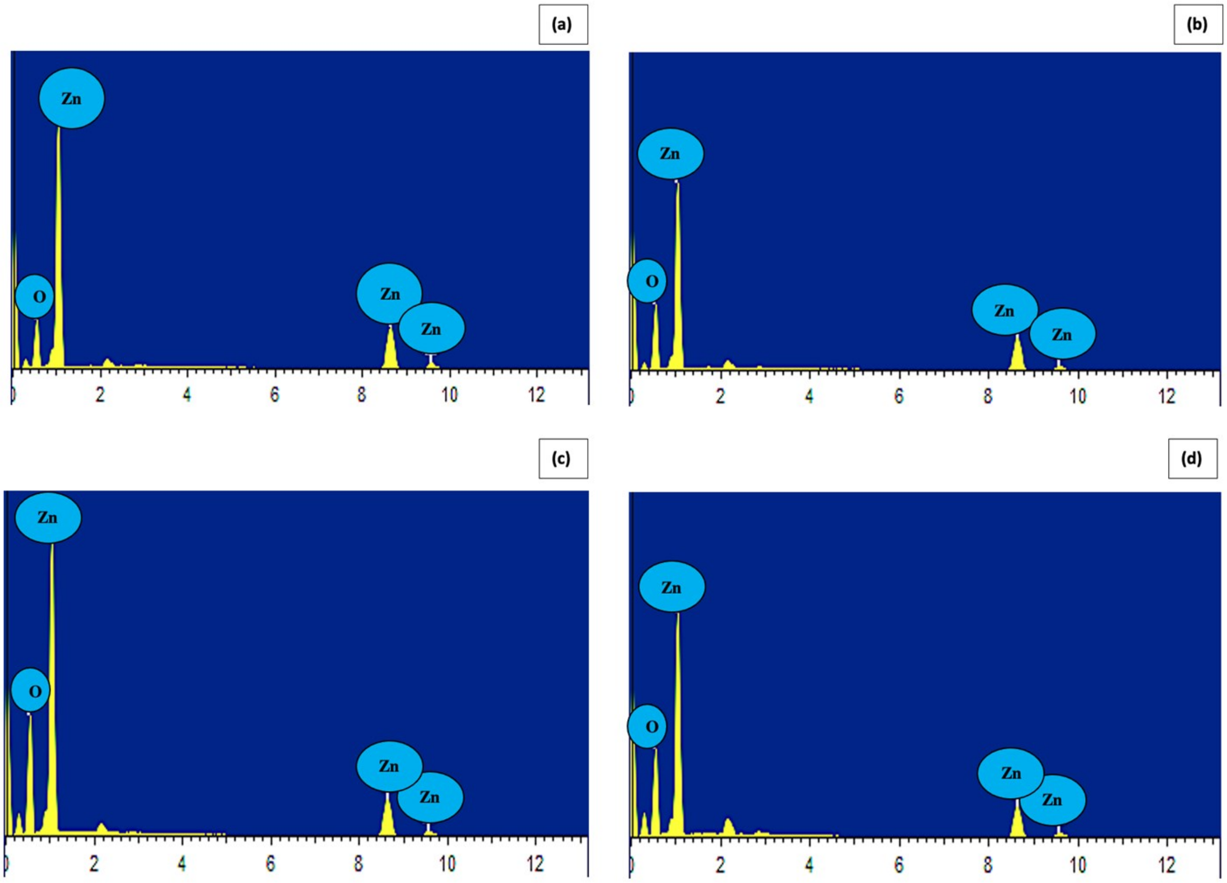

Figure.S2: EDX spectrum of zinc oxide nanoparticles. (a) Star shape, (b) Needle shape, (c) Plate shape, (d) Cubic shape

Table S1: Mean mortality of *Aedes albopictus* mosquito larvae when exposed to different concentrations of four different morphologies of ZnO NPs

Test sample nanoparticles	Concentrations (mg/L)	Mean mortality	LC_{50} (mg/L)	R	Regression equation
Star shape	25<br>50<br>75<br>100	40.0 ± 0.57<br>53.0 ± 0.67<br>60.0 ± 0.57<br>87.0 ± 0.33	38.90	0.807	Y = 1.986X + 1.841
Needle shape	25<br>50<br>75<br>100	40.0 ± 0.57<br>43.0 ± 0.88<br>63.0 ± 0.67<br>67.0 ± 0.88	47.53	0.831	Y = 1.189X + 3.005
Plate shape	25<br>50<br>75<br>100	30.0 ± 0.57<br>43.0 ± 0.88<br>50.0 ± 0.57<br>60.0 ± 0.57	68.38	0.992	Y = 1.230X + 2.743
Cubic shape	25<br>50<br>75<br>100	27.0 ± 0.33<br>40.0 ± 0.57<br>60.0 ± 0.57<br>73.0 ± 0.33	50.24	0.933	Y = 2.653X + 0.487

LC_{50}: lethal dose to kill 50% of the exposed larvae, significant at $P < 0.05$ level; R: correlation coefficient

Table S2: Mean mortality of *Anopheles vagus* mosquito larvae when exposed to different concentration of four different morphologies of ZnO NPs

Test sample nanoparticles	Concentrations (mg/L)	Mean Mortality	LC_{50} (mg/L)	R	Regression equation
Star shape	25<br>50<br>75<br>100	83.0 ± 0.33<br>87.0 ± 0.33<br>90.0 ± 0.57<br>97.0 ± 0.33	4.78	0.886	Y = 1.191X + 4.190
Needle shape	25<br>50<br>75<br>100	70.0 ± 0.57<br>80.0 ± 0.57<br>83.0 ± 0.33<br>87.0 ± 0.33	6.51	0.995	Y = 0.913X + 4.257
Plate shape	25<br>50<br>75<br>100	67.0 ± 0.33<br>76.0 ± 0.33<br>83.0 ± 0.33<br>90.0 ± 0.57	13.64	0.976	Y = 1.378X + 3.436
Cubic shape	25<br>50<br>75<br>100	77.0 ± 0.33<br>83.0 ± 0.33<br>90.0 ± 0.57<br>97.0 ± 0.33	10.47	0.941	Y = 1.624X + 3.343

LC_{50}: lethal dose to kill 50% of the exposed larvae, significant at $P < 0.05$ level; R: correlation coefficient
